# Supplementary material for: Tumor suppressor PALB2 maintains redox and mitochondrial homeostasis in the brain and cooperates with ATG7/autophagy to suppress neurodegeneration
Source: PLoS Genet. 2022 Apr 11;18(4):e1010138. doi: 10.1371/journal.pgen.1010138 (PMC9022806; doi:10.1371/journal.pgen.1010138)
Supplement: S1 Fig — (PDF) [file pgen.1010138.s001.pdf]

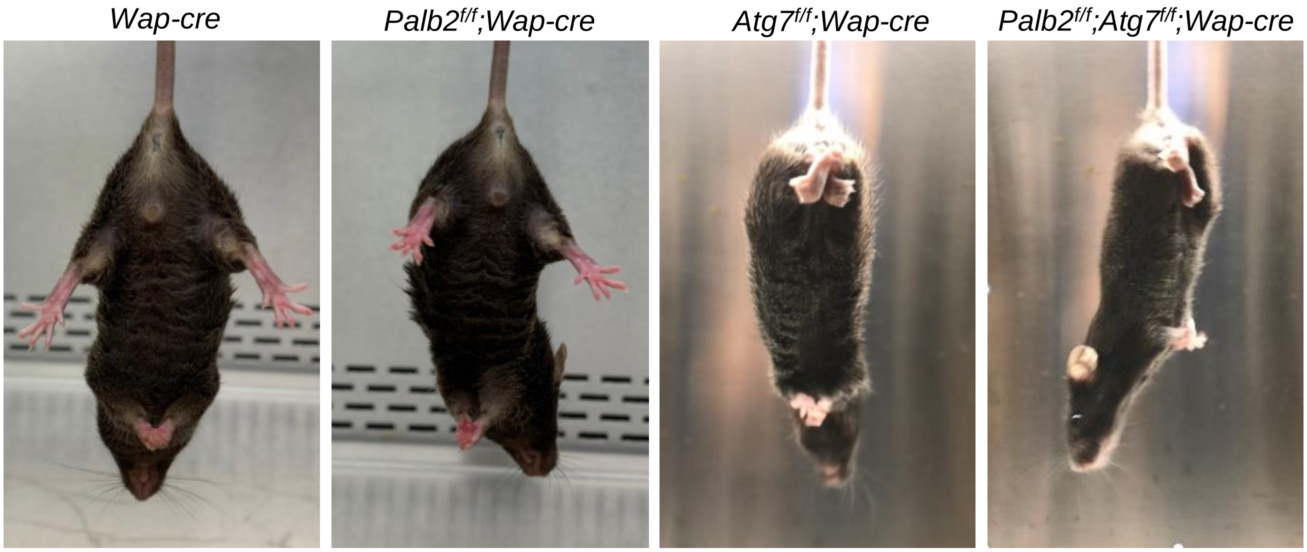

S1 Fig. Hind-leg clasping reflex test of *Palb2*, *Atg7* and *Palb2;Atg7* CKO mice. *Wap-Cre* driven CKO mice at 8 weeks of age were suspended by their tails for 1 min. Clasping was defined as the balling up of one or both hindlimb paws, accompanied by their being pulled into the body and movement of the limbs toward the midline. *Wap-cre* mice and *Palb2*$^{f/f}$*;Wap-cre* mice showed lack of hindlimb clasping (hindlimbs splayed outwards away from abdomen, while *Atg7*$^{f/f}$*;Wap-cre* mice and *Palb2*$^{f/f}$*;Atg7*$^{f/f}$*;Wap-cre* mice showed hindlimb clasping behavior towards the abdomen.
