# Supplementary material for: Tumor suppressor PALB2 maintains redox and mitochondrial homeostasis in the brain and cooperates with ATG7/autophagy to suppress neurodegeneration
Source: PLoS Genet. 2022 Apr 11;18(4):e1010138. doi: 10.1371/journal.pgen.1010138 (PMC9022806; doi:10.1371/journal.pgen.1010138)
Supplement: S2 Fig — (PDF) [file pgen.1010138.s002.pdf]

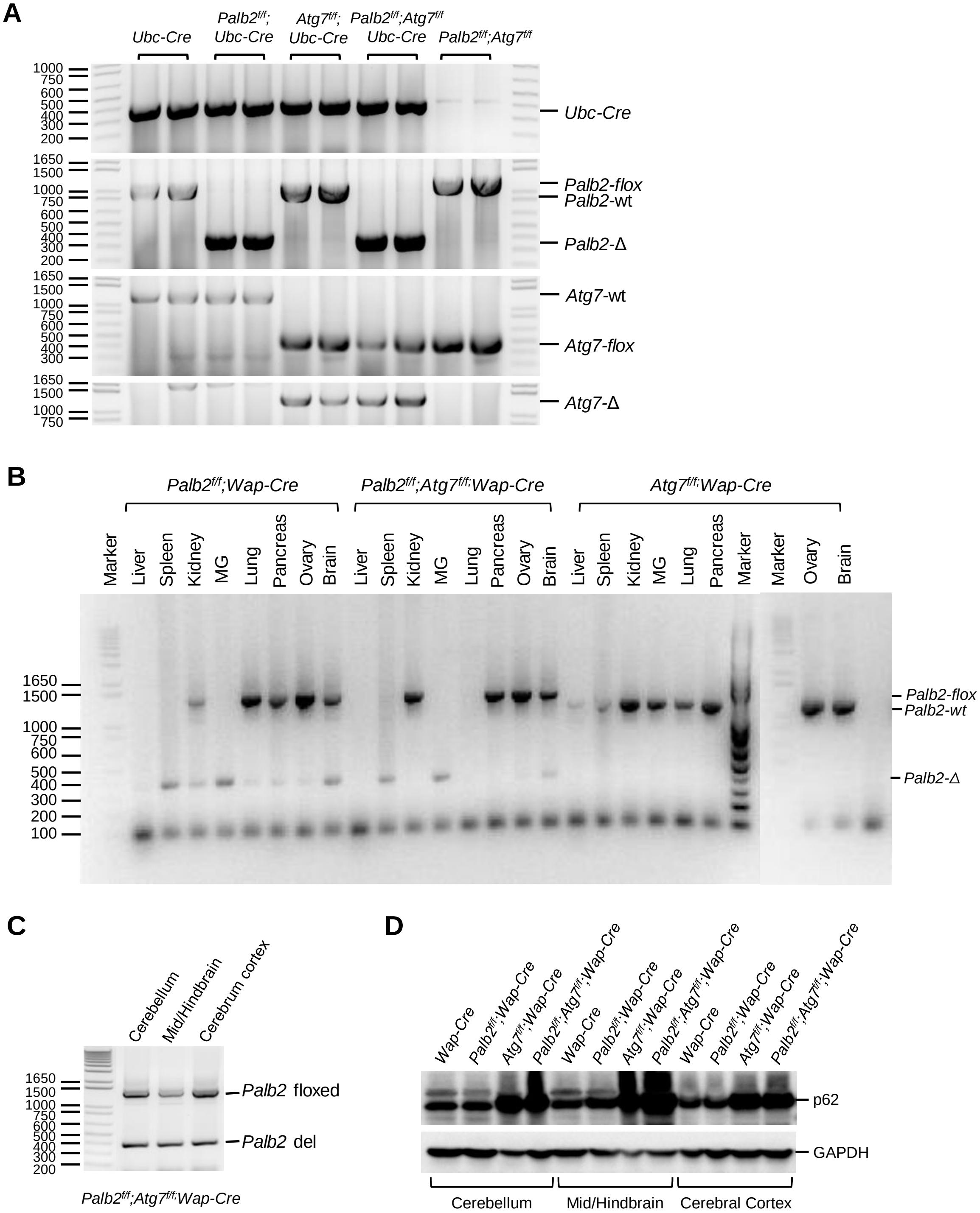

S2 Fig. Detection of *Palb2* and/or *Atg7* deletion in *Wap-cre* or *Ubc-Cre-ERT2* model mice. (A) PCR detection of tamoxifen induced, Ubc-Cre-ERT2-mediated *Palb2* or *Atg7* deletion in the whole brain. **(B)** PCR detection of *Palb2* deletion in different tissues of 6 weeks old *Palb2*$^{f/f}$*;Wap-cre, Atg7*$^{f/f}$*;Wap-cre* and *Palb2*$^{f/f}$*;Atg7*$^{f/f}$*;Wap-cre* mice. **(C)** PCR detection of *Palb2* deletion in different brain regions of *Palb2*$^{f/f}$*;Atg7*$^{f/f}$*;Wap-cre* mice. **(D)** Representative western blots showing p62 accumulation in the cerebellum, mid/hindbrain and cerebrum cortex of 6 weeks old *Wap-cre*, *Palb2*$^{f/f}$*;Wap-cre, Atg7*$^{f/f}$*;Wap-cre* and *Palb2*$^{f/f}$*;Atg7*$^{f/f}$*;Wap-cre* mice. GAPDH was used as a loading control.
