# Supplementary material for: Tumor suppressor PALB2 maintains redox and mitochondrial homeostasis in the brain and cooperates with ATG7/autophagy to suppress neurodegeneration
Source: PLoS Genet. 2022 Apr 11;18(4):e1010138. doi: 10.1371/journal.pgen.1010138 (PMC9022806; doi:10.1371/journal.pgen.1010138)
Supplement: S3 Fig — (PDF) [file pgen.1010138.s003.pdf]

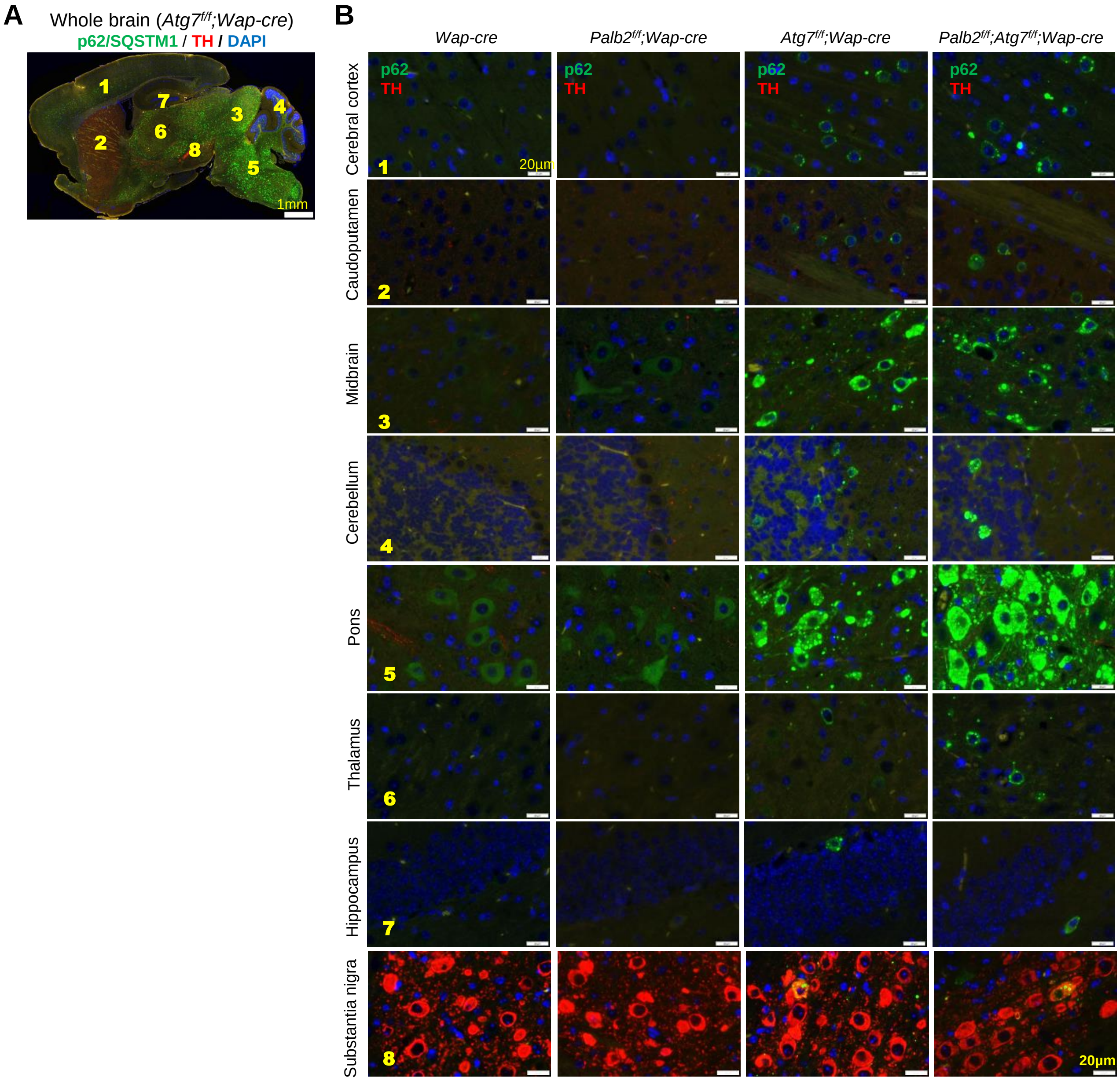

S3 Fig. Expression of p62/SQSTM1 and tyrosine hydroxylase (TH) in the brains of *Palb2*, *Atg7* and *Palb2;Atg7* CKO mice. Midsagittal sections of 6 weeks old brains were analyzed by immunofluorescence (IF), with nuclei stained with DAPI. **(A)** Regions of the brain examined. 1, Cerebral cortex; 2, Caudoputamen; 3, Midbrain; 4, Cerebellum; 5, Pons; 6, Thalamus; 7, Hippocampus; 8, Substantial nigra. A, Scale bar=1mm. **(B)** Representative images of p62 and TH staining pattern in the 8 different regions marked in A. Sale bar=20µm
