# Supplementary material for: Tumor suppressor PALB2 maintains redox and mitochondrial homeostasis in the brain and cooperates with ATG7/autophagy to suppress neurodegeneration
Source: PLoS Genet. 2022 Apr 11;18(4):e1010138. doi: 10.1371/journal.pgen.1010138 (PMC9022806; doi:10.1371/journal.pgen.1010138)
Supplement: S4 Fig — (PDF) [file pgen.1010138.s004.pdf]

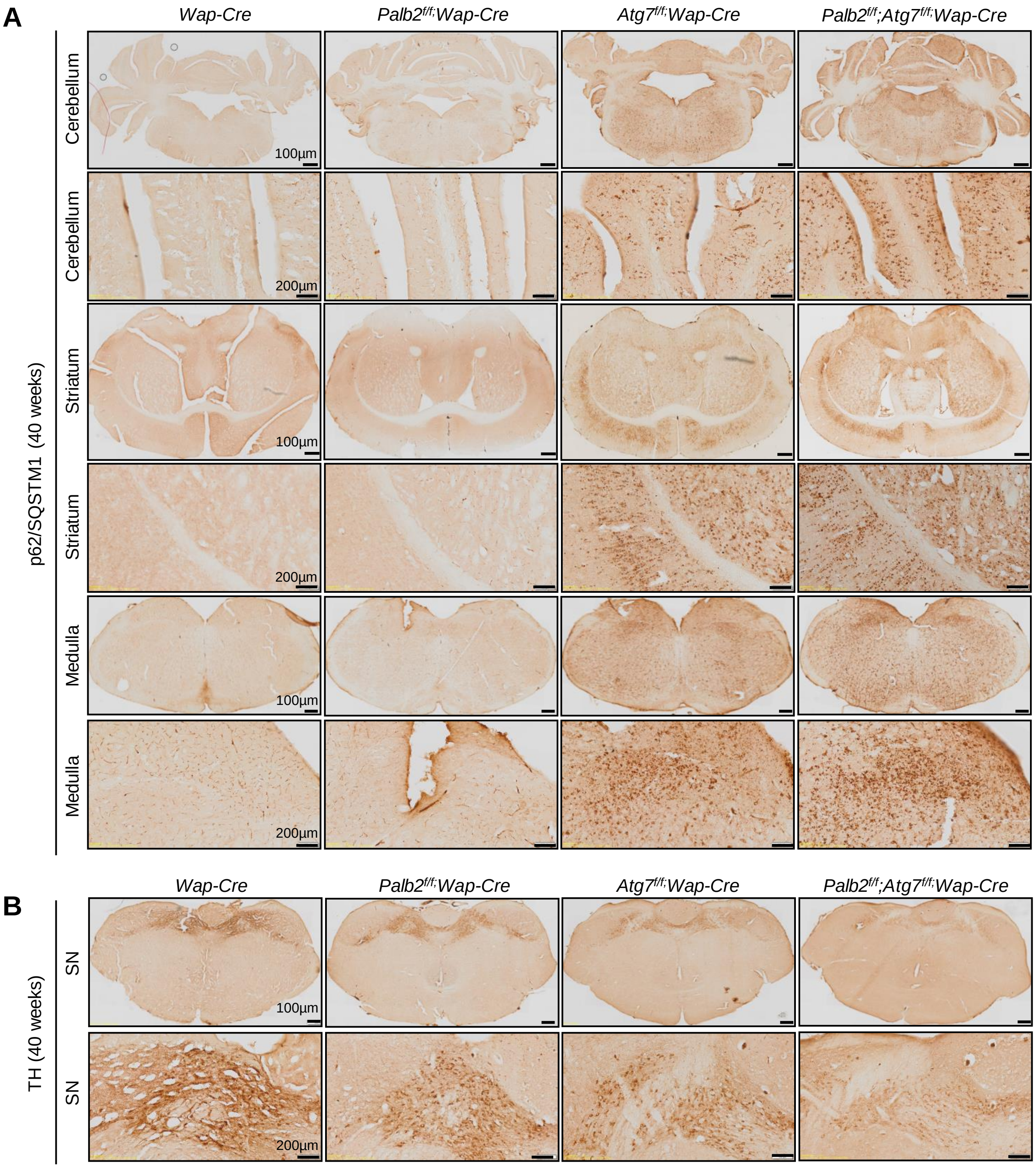

S4 Fig. IHC staining of p62/SQSTM1 and TH in cross sections of the coronal of *Palb2*, *Atg7* and *Palb2;Atg7* CKO mice. **(A)** Representative IHC images of p62 in cerebellum, striatum and medulla of the CKO mice at 40 weeks. Scale bar = 100µm or 200µm as indicated. **(B)** Representative IHC images of TH in the substantial nigra (SN) of the CKO mice at 40 weeks. Scale bar = 100µm or 200µm as indicated.
