# Supplementary material for: Tumor suppressor PALB2 maintains redox and mitochondrial homeostasis in the brain and cooperates with ATG7/autophagy to suppress neurodegeneration
Source: PLoS Genet. 2022 Apr 11;18(4):e1010138. doi: 10.1371/journal.pgen.1010138 (PMC9022806; doi:10.1371/journal.pgen.1010138)
Supplement: S5 Fig — (PDF) [file pgen.1010138.s005.pdf]

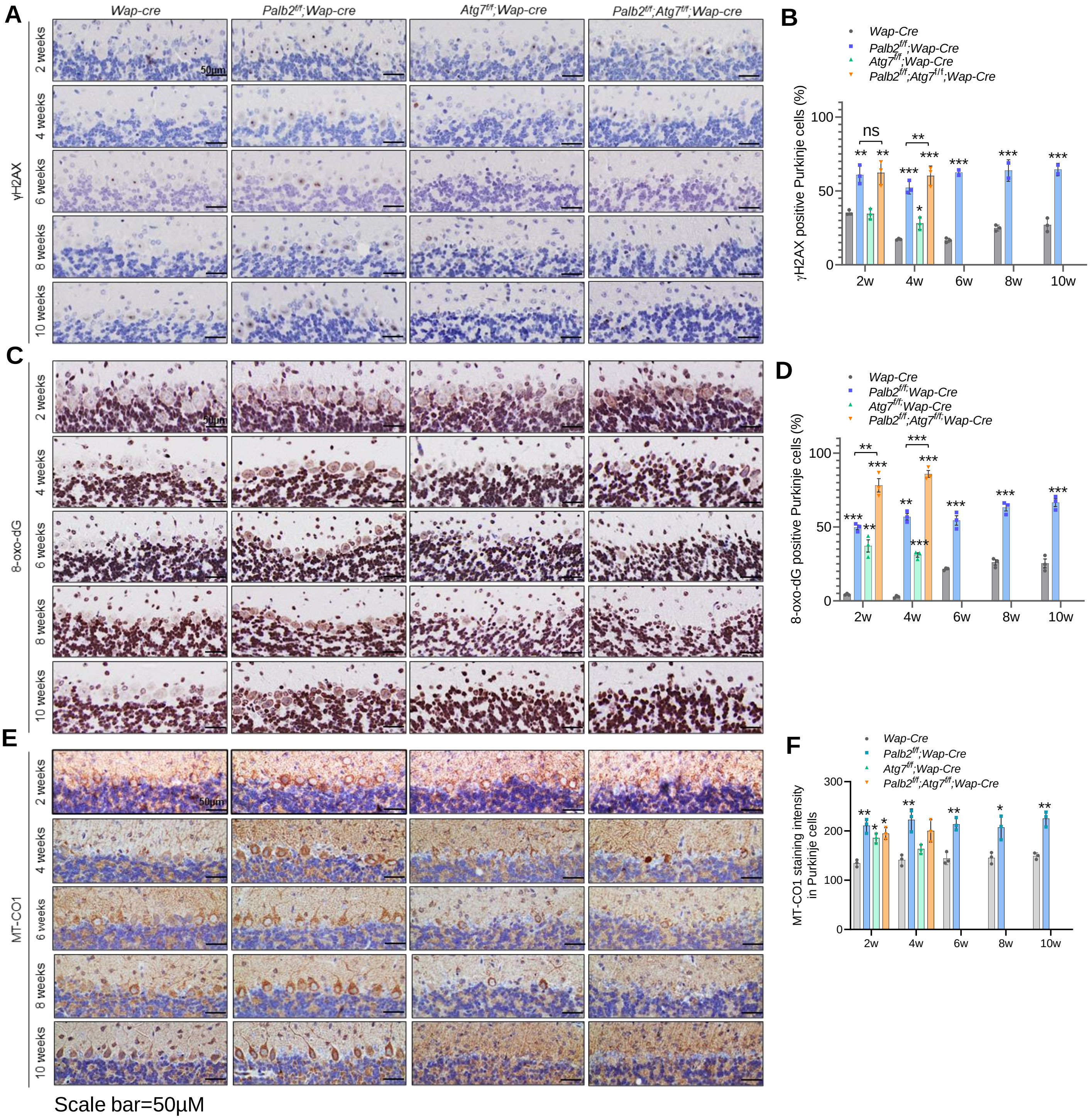

S5 Fig. IHC analysis of markers of DNA damage, oxidative stress and mitochondria in Purkinje cells of *Palb2*, *Atg7* and *Palb2;Atg7* CKO mice. (A-F) Representative IHC images and quantification of γH2AX (A and B), 8-oxo-dG (C and D) and MT-CO1 (E and F) in Purkinje cells of the CKO mice at 2, 4, 6, 8 and 10 weeks of age. Scale bar = 50 μm. n=3, error bars are mean ± SEM. * $p < 0.05$, ** $p < 0.01$, *** $p < 0.001$, unpaired two-sided Student's t test.
