# Supplementary material for: Tumor suppressor PALB2 maintains redox and mitochondrial homeostasis in the brain and cooperates with ATG7/autophagy to suppress neurodegeneration
Source: PLoS Genet. 2022 Apr 11;18(4):e1010138. doi: 10.1371/journal.pgen.1010138 (PMC9022806; doi:10.1371/journal.pgen.1010138)
Supplement: S6 Fig — (PDF) [file pgen.1010138.s006.pdf]

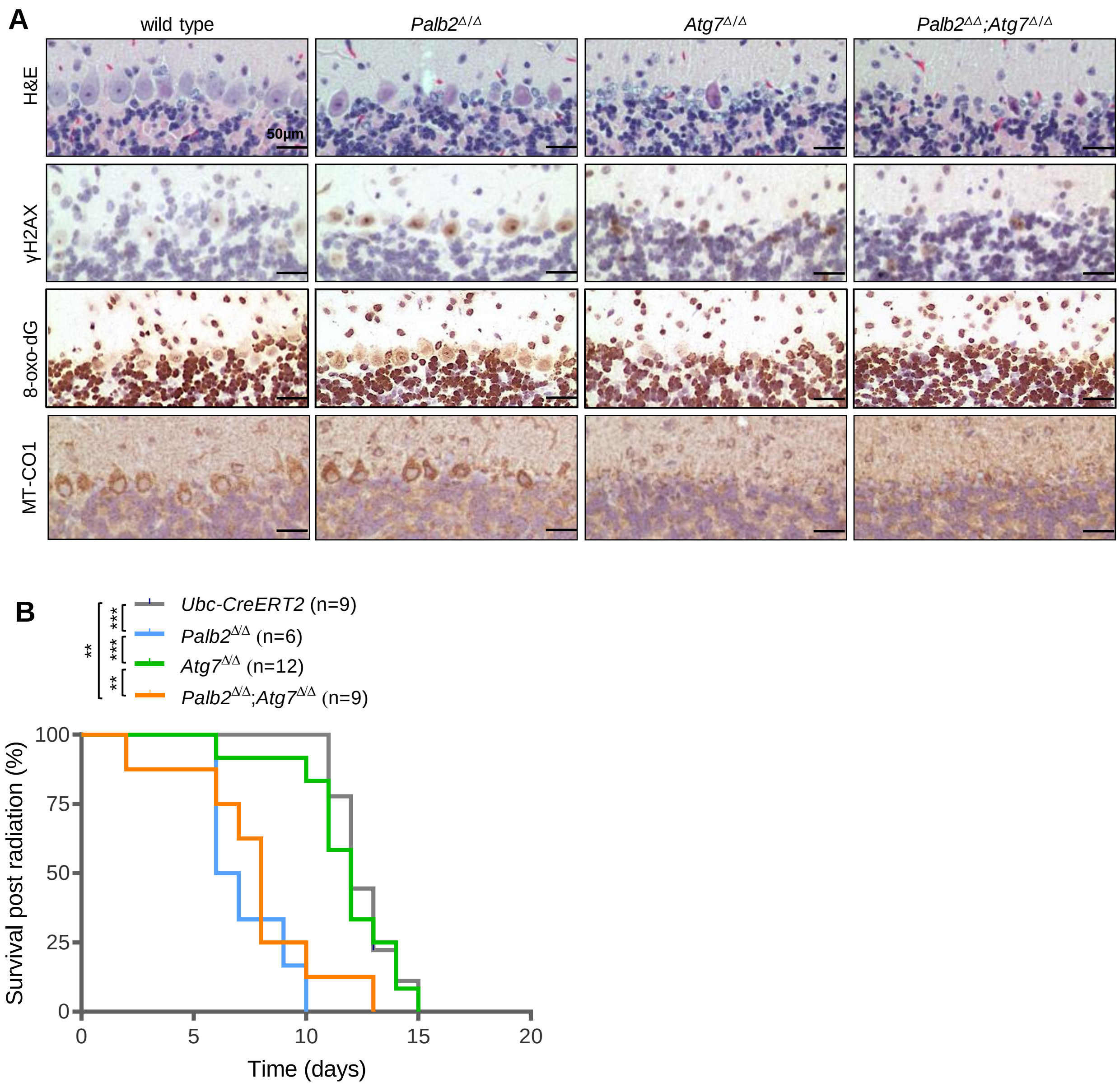

S6 Fig. Markers of DNA damage, oxidative stress and mitochondria in *Palb2*, *Atg7* and *Palb2;Atg7* WBKO mice and the sensitivity of the mice to gamma radiation. (A) Representative H&E staining and IHC images of γH2AX, 8-oxo-dG and MT-CO1 in Purkinje cells and surrounding cells of *Ubc-CreERT2*, *Palb2^{ΔΔ}*, *Atg7^{ΔΔ} and Palb2^{ΔΔ}*;*Atg7^{ΔΔ}* mice. Scale bar=50μm. **(B)** Survival curves of the above mice after 10 Gy of whole-body γ-radiation. **, p<0.01; ***, p<0.001, Log-rank test.
