# Supplementary material for: Tumor suppressor PALB2 maintains redox and mitochondrial homeostasis in the brain and cooperates with ATG7/autophagy to suppress neurodegeneration
Source: PLoS Genet. 2022 Apr 11;18(4):e1010138. doi: 10.1371/journal.pgen.1010138 (PMC9022806; doi:10.1371/journal.pgen.1010138)
Supplement: S7 Fig — (PDF) [file pgen.1010138.s007.pdf]

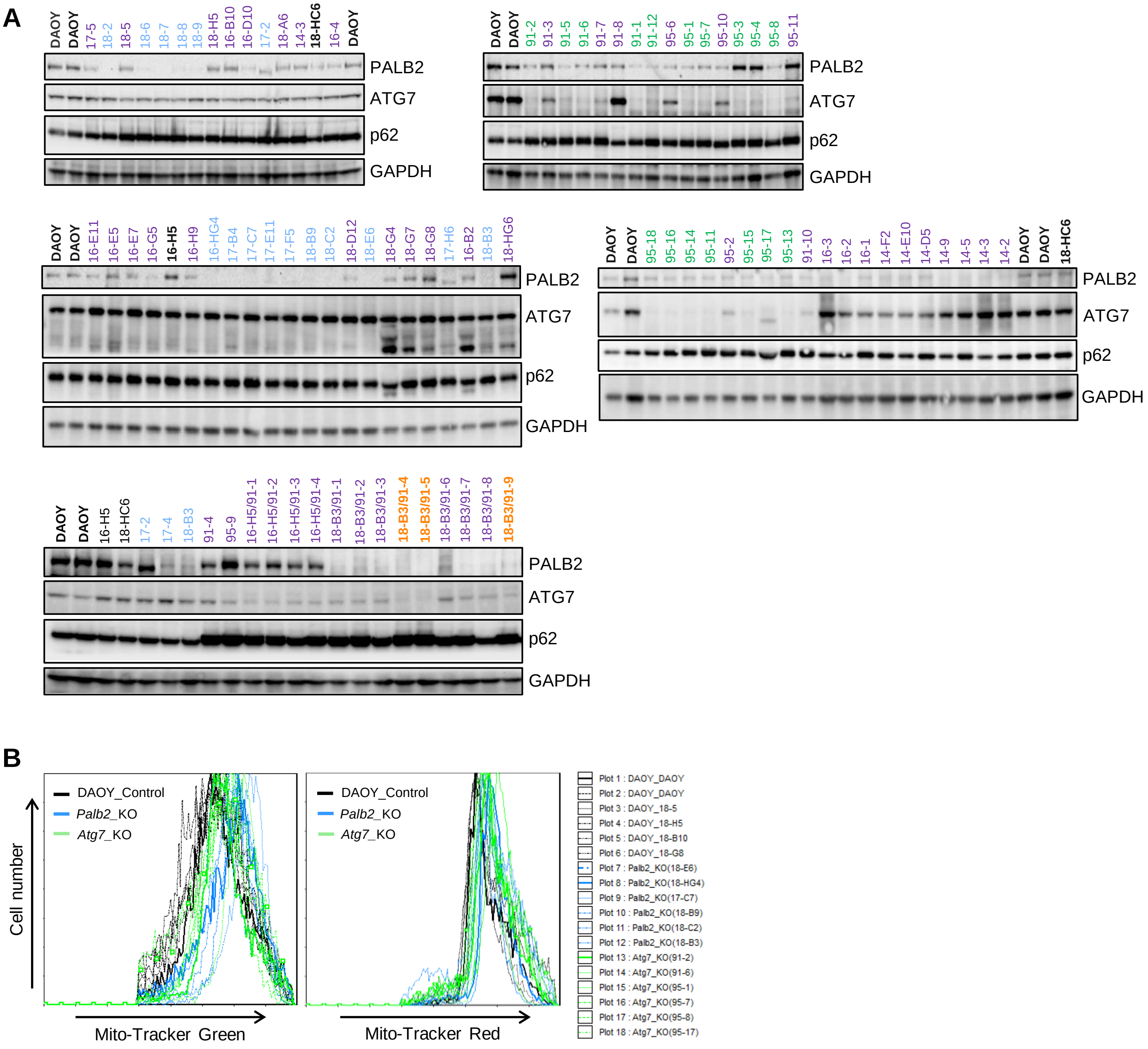


**S7 Fig. Screening of *PALB2*-KO, *ATG7*-KO and *PALB2;ATG7*-DKO DAOY cell clones and measurements of mitochondria in the clones. (A)** Screening and validation of single cell derived *PALB2* and *ATG7* knockout cell clones by western blotting. Clones were labelled by different colors. Bold black, parental or false positive clones that were used as negative controls in mitochondria measurement; Blue, *PALB2* KO clones used for mitochondria measurement; Green, *ATG7* KO cell clones used for mitochondria measurement; Orange: *PALB2* and *ATG7* double KO cell clones. **(B)** Overlay flow cytometry plots of representative MitoTraker Green and MitoTracker Red measurements of mitochondrial mass and membrane potential, respectively. Black, parental DAOY cells and wt control clones; blue, *PALB2*-KO clones; green, *ATG7*-KO clones.
