# Supplementary material for: Tumor suppressor PALB2 maintains redox and mitochondrial homeostasis in the brain and cooperates with ATG7/autophagy to suppress neurodegeneration
Source: PLoS Genet. 2022 Apr 11;18(4):e1010138. doi: 10.1371/journal.pgen.1010138 (PMC9022806; doi:10.1371/journal.pgen.1010138)
Supplement: Attachment — Submitted filename: Huo et al. rebuttal letter final.pdf [file pgen.1010138.s021.pdf]

Response to Reviewers' Comments

We thank the reviewers for their critical review and constructive comments. We have made our best effort to address all of the comments in the revised manuscript. Below is our point-to-point response to the comments.

Reviewer #1: In this manuscript, Huo et al reported the role of PALB2 (a tumor suppressor that is known to be essential for DNA repair, redox homeostasis and breast cancer suppression) and ATG7/autophagy in the suppression of neurodegeneration in mice. Using two types of Cre mice (Wap-Cre [leaky expressing Cre in neurons] and Ubc-Cre-ERT2), the authors found that co-deletion of Palb2 and Atg7 causes severer neurodegeneration and earlier lethality compared to single KO mice. Pathological studies revealed the enhanced DNA damage, oxidative stress, mitochondrial dysfunction and apoptotic cell death, especially in Purkinje cells, in double KO mice compared to single Ko mice, suggesting that Palb2 and Atg7 may synergistically function to suppress these abnormalities. The cellular phenotypes were recapitulated in double KO medulloblastoma DAOY cell lines. Mechanistically, the authors demonstrated that further loss of p53, but not Braca2 (a critical factor for DNA repair) partially rescued the phenotypes (lethality, Purkinje cell loss, and abnormal behaviors) of double KO mice at least to the levels of Atg7 KO mice. Survival rates of double KO mice were also partially rescued by treatment with NAC, a ROS scavenger, suggesting that high levels of oxidative stress rather than increased DNA damage contributed to the lethality of double KO mice. Taken together, the authors concluded that PALB2 maintains redox and mitochondrial homeostasis in the brain cooperatively with Atg7.

Although the critical role of autophagy in protecting against oxidative stresses is already well established and it is easy to assume that deletion of two different and important pathways can cause severe phenotypes, this study for the first time demonstrates the genetic interactions between PALB2 and autophagy in the brain. To support their conclusion, however, there remain some concerns that need to be clarified.

1. In Figure 7, the causal relationships between the higher oxidative stress and the severer phenotypes of double KO mice are unclear. The authors should confirm the effect of NAC on neurodegeneration, oxidative stress, and DNA damages by histological analyses. The effect of NAC could also be checked using DAOY cell lines.

Thanks for this suggestion. We have conducted the suggested experiment using the whole-body double KO mice. Basically, we treated *Palb2*$^{f/f}$*;Atg7*$^{f/f}$*;Ubc-CreERT2* mice with NAC for 8 days starting from the first day of 4-daily TAM injections (to delete the genes) and then analyzed their brain by IHC. We found that NAC treatment strongly rescued Purkinje cell death (new Figure 7D and E). Moreover, NAC treatment significantly reduced oxidative stress in midbrain neurons but produced no significant difference in γH2AX staining, which marks DNA double strand breaks. Note that Purkinje cells had been mostly lost in NAC untreated mice, therefore we didn't compare γH2AX and 8-oxo-dG in them. This set of results strongly support the idea that oxidative stress is a major cause of neurodegeneration in the double KO mice.

We also tried treating the DAOY cells with NAC; however, despite multiple attempts with different concentrations, we were unable to detect any reduction in ROS levels after NAC

treatment. In fact, ROS levels often went up after NAC treatment, which could be due to activation of certain metabolism-related signaling pathways in the cells.

2. The authors should discuss or provide further evidence about the following points: (1) how Palb2 regulates redox and mitochondrial homeostasis, (2) how p53 causes severer phenotypes, (3) whether autophagy-independent functions of Atg7 are involved or not.

Thanks for the suggestions.

1) In terms of redox regulation per se, we have previously reported that PALB2 directly interacts with KEAP1, a negative regulator of the master antioxidant transcription factor NRF2, thereby protecting NRF2 from KEAP1-mediated degradation and nuclear export. As for mitochondrial homeostasis, our newly added RNA-seq results showed broad upregulation of mitochondrial genes in PALB2 knockdown DAOY cells (new Figure 4). This result suggests that PALB2 may act as a repressor of a master regulator of mitochondrial gene transcription either by directly interacting with the transcription machinery or by altering chromatin structure, possibly in association with its close interaction partner MRG15, which is a component of the NuA4 chromatin remodeling complex. The other possibility is that the upregulation of mitochondrial gene expression and biogenesis is an adaptive response to increased ROS upon loss of PALB2. Increased mitochondria with suboptimal quality, in turn, may also contribute to increased ROS in PALB2 depleted or KO cells. These have been added to the discussion.

2) As for why p53 causes severer phenotypes, we think it is mainly due to apoptosis-inducing activity, as deletion of *Trp53* reduced apoptotic neurons in the cerebellum of the double *Palb2*;*Atg7* double CKO mice. We have added a dedicated paragraph discussing p53. Please see revised discussion section.

3) As to the autophagy-independent functions of ATG7, we find it difficult to discuss since in this study we mainly focused on autophagy-related parameters, namely DNA damage, redox and mitochondrial regulation, as well as DNA damage. Still, we acknowledged this issue at the end of the discussion.

Reviewer #2: PALB2 is a tumor suppressor protein associated with familial breast cancer and is implicated in DNA repair and redox homeostasis. In the present study Huo et al. report that Palb2 deletion in the mice brain (driven by Wap-Cre) or in the whole-body (driven by Ubc-Cre-ERT2) lead to motor deficits and its co-deletion with the essential autophagy gene Atg7 accelerates and exacerbates neurodegeneration that was initially induced by Atg7 deletion. Such a co-deletion led to loss of Purkinje cells (controller neurons of motor coordination and balance) accompanied by increased DNA damage, oxidative stress and mitochondrial dysfunction. The effect of mitochondrial dysfunction and oxidative stress was verified in PALB2 KO human DAOY medulloblastoma cells. To rull out the possibility that the DNA damage activity of PALB2 is involved in the authors have generated a double knockout mouse in which BRCA2 was deleted together with ATG7. Such a deletion led to a delay in neurodegeneration induced by ATG7 and therefore they concluded that the exacerbating effect of neurodegeneration in Palb2;Atg7-CKO mice is not caused by the loss of DNA repair function of PALB2. Finally, a triple KO mouse was created in which p53, Palb2 and Atg7 were co-deleted, leading to partial recovery the neurodegeneration phenotype. This led the authors to conclude that the overall neurodegeneration caused by the lack of Atg7 and PALB2 is under the control of p53.

While the body of work invested in this study is well appreciated, the main conclusions and the authors' model are not fully supported by the data, which mostly remain too preliminary. Almost every claim the authors are making requires additional complementary experimental evidence which are missing throughout. For example, the new role for PALB2 in the regulation of mitochondrial biogenesis and function and that this role leads to exacerbating effect of neurodegeneration in the absence of ATG7 should be better characterized.

Thanks for your appreciation of the body of work invested and the critical evaluation of our manuscript. We agree that much remains to be done to directly prove several aspects of our proposed model, therefore we have decided to remove it. In the revised manuscript, to substantiate our results on PALB2 regulation of mitochondria, we have added RNA-seq and qRT-PCR results showing that loss of PALB2 leads to broad upregulation of mitochondrial gene expression (new Figure 4). To further confirm that excessive oxidative stress is the main reason for the severe neurodegenerative phenotypes in the double KO mice, we conducted NAC treatment followed by IHC analysis as suggested by reviewer #1, and the results (new Figure 8 D-E) strongly supports the conclusion.

The authors ignore the role of autophagy in the regulation of DNA damage which seems important here.

In the original manuscript, we were mainly focused on cerebellum and Purkinje cells, because the motor deficits at young ages that we measured in this study are best explained by Purkinje cell loss. In these cells, we did not observe any discernible increase in γH2AX staining upon loss of ATG7 (before the cells were lost). We have now added results from midbrain, in which neurons clearly showed increased γH2AX foci formation (new Figure 3A, B).

This is also relevant to the conclusion that p53 indeed plays a role in this process. Is it a direct involvement? Which of the downstream factors are needed here? These and many more questions remain unanswered.

We think p53 does play a role in Purkinje cell loss and motor deficits in the *Palb2*;*Atg7* double CKO mice, as its loss substantially rescued the Purkinje cells loss and almost fully restored the beam balance score of the mice. It is not very likely that p53 induction in *Atg7*-deleted Purkinje cells was caused by DNA damage for the same reason above, although it may still contribute. Moreover, p53 loss appeared to only rescue the added severity caused by *Palb2* ablation in the double CKO mice and failed to rescue the Purkinje cell loss and bean balance deficit of *Atg7* single CKO mice. We agree that there remain open questions that need to be addressed, and we hope to address them in future studies.

Additional comments:

1. It is well characterized that the loss of ATG7 leads to neurodegeneration, but the authors indicated that: "Unexpectedly, Atg7-CKO mice showed progressive motor deficit….suggestive of cerebellar involvement".

Wap-Cre was designed to have Cre recombinase directed to secretory mammary epithelial cells and to generate conditional knockouts for studying breast cancer and lactation. Although leaky expression had been reported in the brain, we did not expect such strong motor deficits (due to highly efficient deletion of *Atg7* in the brain). However, we agree that the wording may be confusing, and we have now added "Wap-Cre driven (Atg7-CKO)" into the text.

2. The authors claim that PALB2 exacerbates neurodegeneration induced by ATG7 loss, but they did not test any markers of neurodegeneration additionally to Purkinje cells survival.

Because the whole phenotype is very complex and, in our view, extremely difficult to fully address in one manuscript, we mainly focused on motor deficits at young ages and Purkinje cells because most of the early phenotypes of the mice can largely be explained by loss of this cell type. Other than Purkinje cells, we also conducted TUNEL to detect apoptosis. Additionally, we also attempted to check a-Synuclein by IHC, however we were unable to obtain specific signals with the antibody used.

3. Likewise, the requirement of PALB2 for mitochondrial homeostasis was hardly investigated and the presented results are not convincing. Moreover, the claim that “PALB2 leads to increased mitochondrial biogenesis” was not explored at all (real time PCR of mitochondrial genes or any other method).

Thanks for the suggestion. We have now added RNA-seq results that show broad upregulation of mitochondrial genes in DAOY cells depleted of PALB2. Several genes were also validated by qRT-PCR. Please see new Figure 4 and related text.

4. The effect of p53 deletion was not deeply explored and its role in the partial recovery of the Palb2;Atg7;Trp53-CKO mice survival remains mostly not clear.

We see that p53 deletion largely rescued the effect of *Palb2* deletion, both in Purkinje cell number and the number of apoptotic cells in the cerebellum, so that the triple CKO mice behaved similarly to *Atg7* single CKO mice in the setting used.

5. Figure 1A – survival discrepancy of Atg7-CKO mice between the text (604 days) and the figure (596 days).

Corrected. Thanks for catching this error.

6. The Atg7-CKO mice showed limb clasping reflexes when suspended by the tails, but for an unclear reason that data was not shown What about Palb2;Atg7-CKO?

Similar to *Atg7*-CKO mice, these double CKO mice also showed limb clasping reflexes when suspended by the tails. Pictures of representative mice are now provided in new Supplementary Figure 1.

7. Figure 2A – the staining for tyrosine hydrolase (TH) a marker of dopaminergic neurons does not contribute to this figure and may be presented in supplementary figures. Instead, it may be more productive to co-stain any other neurodegenerative markers together with p62.

We agree that TH staining does not contribute in the original context. In the revised manuscript, in response to a comment from review #3, we added an additional set of images showing progressive p62 accumulation in TH positive neurons at 6 and 10 weeks and significant loss of these neurons in the substantia nigra of the double CKO mice at 40 weeks. Please see new Figure 2C, 2D and Supplementary Figures 3 and 4.

8. Figure 2D – the TUNEL staining for 8 weeks should be presented to be in line with 2E.

Since there was no significant difference between *Atg7*-CKO and *Palb2*;*Atg7*-CKO at 8 weeks, we have removed 8 weeks from the bar graph in the original Figure 2E (now 2H), rather than adding the images, due to space limitation in Figure 2 (it is already a whole page).

9. Figure 3 – all images for all time points should be presented (at least in supplementary material). The high signals in Atg7-CKO and Palb2;Atg7-CKO after six weeks described in panels 3B and 3D is not clear given that apoptosis already occurred. The explanation in the text is unclear.

We have updated Figure 3, which now shows only one time point for cerebellum/Purkinje cells, with all images for all other time points moved to or added in Supplementary Figure 5. Additionally, we added images from the midbrain in Figure 3 as noted above. Quantifications of midbrain neurons are also added in Figure 3.

10. Figure 4F and 4G – the measurements of total mitochondria and mitochondrial membrane potential only by mitotrackers immunofluorescence are not sufficient, should be validated at least by FACS.

Figure 4F (5F in revision) are representative fluorescence images, and results in Figure 4G (5G in revision) were indeed measured by flow cytometry (or FACS).

11. Figure 4H and 4I – the OCR measured by seahorse represents only 4 cell lines, but the mitochondria parameters of that measurements (I) show 8 cell lines. These two panels should be in line.

We have now added the other four cell lines and presented the data in two separate graphs within the same panel (new Figure 5H).

In addition, the authors claim that the double knockout DAOY medulloblastoma cells showed strong defect in mitochondrial respiration due to spontaneous death of DKO, but may be 100 minutes are not enough for DKO necrosis\apoptosis and they just switch to glycolysis?

What we wrote was “These data suggest that a strong defect in mitochondrial respiration may be a major contributor to the spontaneous death of DKO cells.” We agree that 100 minutes are probably not enough for significant necrosis/apoptosis to occur.

12. Figure 6 – a plot of cause of death (%) is missing.

Such a plot has been added (6C in revision). Thanks.

13. Figure 7A and 7B – the recovery phenotype by ROS scavenger is more relevant to figure 3. Panels C and D are not contributing since the model is too preliminary and requires additional investigation.

As noted before, the proposed model in the original Panel D has been removed. We feel that the summary in the original Panel C should give reader a quick and clear recap of the data presented, therefore we hope it is acceptable. Thanks for your understanding.

14. The authors decided to start the results section trying to “…to further examine the role of

autophagy in mammary tumor development…" but moved to determine neurodegenerative effects since they used C57BL\6 genetic background which is resistant to mammary tumorigenesis.

We agree that C57BL/6 is more resistant to mammary tumorigenesis than some other genetic backgrounds such as FVB. At the beginning of our study, the *Atg7* conditional allele was only available in pure C57BL/6 background, and it would take significant amount of time to backcross. Our own experience was that although C56BL/6 delays mammary tumorigenesis, it would not prevent it from happening. At the same time, the longer time frame may allow detection of more subtle differences, especially when p53 is co-deleted. Therefore, we took the risk. What we found in the end is that mammary tumorigenesis in *Brca2*-CKO mice was indeed strongly suppressed (in p53 wt setting).

15. The discussion section is mostly a rewriting of the results and it suffers from lack of relevant references.

Thanks for pointing this out. We have deleted several descriptive passages, rewritten some other parts, and added two paragraphs discussing p53 and how PALB2 may regulate mitochondrial homeostasis. More references have also been added.

Reviewer #3:

In this manuscript, the role of the tumour suppressor Palb2 on neurodegeneration is studied, especially regarding its loss in synergy with the loss of the essential for autophagy gene Atg7. It is shown that loss of PALB2 accelerates and exacerbates cerebellar Purkinje cell degeneration observed in Atg7 KO animals resulting in motor function deficits and reduced survival, and that this PALB2 action is exerted at least partially through p53 activity. The data reveal a role of PALB2 on mitochondrial function and reinforce existing data on the role of PALB2 in redox homeostasis, which could contribute to neurodegeneration observed. Partial rescue of survival after treatment of double KO animals with a ROS scavenger confirms the role of ROS accumulation in reduced survival. Moreover, it further suggests disturbed redox homeostasis as a mechanism contributing to the exacerbation of neuronal degeneration observed in Atg7 KO animals, upon PALB2 loss. This is a manuscript with interesting findings, and a wide set of, in large, convincing experiments. Nevertheless it suffers in results presentation, as well as in text structure and phrasing. Moreover, a small set of the data presented should be further analyzed. We suggest the following changes before publication:

Thanks for your very careful review and overall positive evaluation of our manuscript.

Major

1. The results and figures sections have to be more carefully crafted, as the following mistakes are recurrent:
a. Describe with clear distinction between observations of staining intensity and descriptions of the quantification of IHC stainings. Lack of clear separation between the two is a recurrent problem throughout the results text (e.g. results paragraph "Requirement of PALB2 for mitochondrial homeostasis in the brain").

It is true that IHC staining results were sometimes described in intensity terms and sometimes in positivity terms. There is a reason for that. For example, MT-CO1 staining was seen in all Purkinje cells and the pattern was more uniform, therefore we only compared and described intensity. In contrast, γH2AX was only seen in some cells and the pattern was often hard quantify, so we mainly compared positivity, or the percentage of positive cells. When there was clear difference in intensity, we would also mention that. For 8-oxo-dG, the situation was in between. Thanks for your understanding.

b. In results, page 7, authors describe differences observed in p62 IF stainings, without mentioning the age in which these differences are observed. This happens again during the results regarding 4-HNE staining (results, pages 9-10). Mouse ages should be found on every result description throughout the results section.

Mouse ages have been added in all applicable places.

c. While one finds results from experiments on multiple mouse ages in the relevant figures, these results are not described in the text, e.g. description of the results of in vivo MT-CO1 stainings for weeks 6-10 is missing from the text (results page 10), while description of the results from quantification of the SOD2 staining is completely missing from the same paragraph. Description of the p53+ Purkinje cell quantification from results, page 14, for ages 8-10 is also not provided. These and all results presented in the figures should be described in more detail in the results text.

Thanks. In the revised manuscript, we have made our best effort to describe all figure panels.

d. Often, in the figures presenting data, quantification results from several mice ages are found in the graphs, but no representative images are shown for all the mice ages (2B-C, 2D-E, 3A-B, 3C-D, 3G-H, 3I-J, 6D-E). It would be more informative if images from all ages presented in the graphs would also be shown in the figures.

In Figure 2, images from all ages that are not in the main figure are now presented in Supplementary Figure 5. For Figures 3A-B, 3C-D, 3H-I and 3J-K, we revised them to include only one representative age for cerebellum while adding midbrain at the same age. For Figure 6D-E, we removed the later time points as Purkinje cells had been most lost by then in *Atg7* and *Palb2*;*Atg7* CKO mice (7E in revision).

e. Moreover, the main text references to the figures should be accurate. For example, in page 14, the reference to Figures 6F and G should change to refer to only figure 6G.

Thanks. This has been corrected.

2. In results, page 5, T50 of the Atg7-CKO mice is reported to be 604 in the text and 546 in Figure 1A. Please correct this discrepancy.

Thanks. This has been corrected.

3. Supplementary figure 1A should be better explained. Why do Palb2f/f;Atg7f/f Ubc-Cre and Atg7f/f Ubc-Cre mice have both Atg7-Δ and Atg7-flox bands? Is there no way of telling through PCR wt Palb2 from flox Palb2 allele? Also, the Atg7-wt bands are not very convincing.

1) *Atg7-flox* bands in *Palb2*$^{f/f}$*;Atg7*$^{f/f}$ *Ubc-Cre* and Atg7$^{f/f}$ Ubc-Cre mice may due to incomplete *Atg7* deletion in this particular experiment. In a new experiment shown in new Fig 8C, we saw virtually complete deletion after TAM treatment.

2) For *Palb2*, there was a mistake in the original figure, the band labelled as *Palb2*-flox should be wt and wt should be flox. Thanks for catching it.

3) For genotyping of *Atg7* by PCR, the *Atg7-wt* band should indeed be larger than *Atg7-flox* band, which is due to the fact that the floxed allele has a significant part of the intron deleted (PMID 15866887).

4. Regarding p62 IF, the difference is obvious from the images in the age of 10 week old mice, but not in 6 week old mice (if any). In general images and specific brain areas depicting p62 IF should be in higher magnification.

Thanks for the suggestion. Images in Figure 2A have now been brightened so that the differences are easier to see. Higher magnification images of different brain areas are now provided in Supplementary Figure 3.

5. Regarding tyrosine hydroxylase IF, a higher magnification of the substantia nigra, or quantification of the positive cells in the substantia nigra would be necessary.

Higher magnification images of the substantia nigra are now provided in Figure 2C and Supplementary Figure 4B. Figure 2C shows p62 accumulation in TH positive neurons at 6 and 10 weeks, and Supplementary Figure 4B shows substantial loss of TH positive neurons at 40 weeks in the double CKO mice.

6. Regarding 4-HNE staining. This is a result that is found useful by the authors in data interpretation later on in the manuscript; it should, thus, be more substantiated with quantification, and not based only on observation.

Please see Figure 3F for quantification based on intensity.

7. Statistical analysis for results in several images is missing (3H, 3J, 5D, 6E, 6I).

Added. Thanks.

8. Discussing experiments regarding treatment of the Ubc-Cre-ERT2 model animals with N-Acetyl-Cysteine, you find that NAC treatment prolongs survival of Palb2;Atg7-WBKO mice and suggest that NAC protects against late death cause, which probably is neurodegeneration. Are there motor coordination data from NAC-treated animals such as delay of neurological symptoms onset, or IHC data regarding Purkinje cell survival supporting this suggestion?

We have generated additional mice and conducted the IHC following NAC treatment. Indeed, the severe loss of Purkinje cells in *Palb2*$^{\Delta/\Delta}$*;Atg7*$^{\Delta/\Delta}$ mice were greatly rescued by NAC treatment. Please see new Figure 8D and 8E. Thanks for the suggestion.

9. In the materials and methods section authors describe a preparation of tissues for electron microscopy to measure mitochondrial mass, but relevant results are not discussed in the results section and in figures. It would be greatly adding to the manuscript to present this data.

In an earlier draft, we included the electron microscopy data but then deleted it prior to submission, because we only have 2 mice/specimen for each genotype, and it is also hard to be sure that we are looking at the same area of the rather heterogenous brain tissue. The data, however, indeed point to increased mitochondrial biogenesis in the midbrain of *Palb2*-deleted mice. This part of the methods section has been deleted. Thanks for the understanding.

10. In the materials and methods section authors mention that they used male KO mice for neurodegeneration and female for carcinogenesis. Please explain your thinking in more detail. Where no female mice used for any neurodegeneration study and motor assessment?

This was done primarily to reduce the number of mice used and the labor and costs associated. Since our primary research area is breast cancer, which can only be studied with females, using the males generated in the breeding to study neurodegeneration would to be a very good use of the animals. Additionally, we were also concerned that the parameters we studied, i.e., DNA damage, oxidative stress and mitochondrial activity, may be influenced by the female hormonal cycle, which is not easy to control.

11. In results, page 16, authors mention: “Our studies also establish PALB2 as a suppressor of neurodegeneration in its own right, representing another new function of PALB2”. As by itself loss of PALB2 does not affect neurodegeneration in a great extent, this statement while not false, should be downtoned.

The sentence has been deleted.

12. In the results concerning BRCA2 loss, page 13, authors mention: “As shown in Figure 5F-I, γH2AX positivity in Purkinje cells of Brca2-CKO mice was slightly lower than that in Palb2-CKO mice but still much higher than that in control mice; in contrast, their 8-oxo-dG and MT-CO1 staining signals were substantially weaker than those in Palb2- deleted Purkinje cells“. In this case, the results show that a statistically significant smaller number of Purkinje cells from Brca2-CKO mice are positive for γH2AX and 8-oxo-dG in comparison with the Palb2-CKO mice, while a statistically significant higher number of Purkinje cells from Brca2-CKO mice are positive for γH2AX and 8-oxo-dG in comparison to the Wap-cre mice, and this is similar for both markers. Thus, a comparison of positivity levels between these markers cannot lead to safe conclusions. The same goes for MT-CO1 marker. Also, comparing the results coming from staining intensity quantification (MT-CO1) to results coming from cell positivity measurements seems to be a stretch. Please rephrase.

As mentioned before, the reason why we described the results in such a way is that positive γH2AX and 8-oxo-dG staining was seen only in some cells, whereas MT-CO1 was seen in all cells. Therefore, we described γH2AX and 8-oxo-dG signals in terms of positivity and sometimes also intensity (where appropriate to us), but for MT-CO1 parameters we only used intensity. Sometimes it is very hard to describe the staining pattern and we have to let readers themselves to appreciate the subtlety from the images, which is certainly a limitation of this type of analyses. Thanks for your understanding.

13. Annexin V assay is not described in the methods, the data is not but mentioned in the results text, and there is no statistics shown. Has this experiment been duplicated? Please provide all relevant information in the methods section, and explain in detail the data in the results section.

Thanks for pointing this out. The assay was done in 3 independent experiments, each in technical duplicates. This information has been added to the figure legends and the method has also been added to m Methods section.

14. Accurate number of animals used, as well as times of experiment repeated should be mentioned in detail for all experiments and each group and time point. Also, it is recommended to have at least three animals in each group examined so statistical analyses results are robust.

We have thoroughly revised figure legends, which now contain numbers of mice used. We had 3 or more mice for all experiments presented except IHC of *Trp53*-deleted mice, for which we only had 2 mice available, which we regret. Fortunately, the two mice gave nearly identical results that are also consistent with the survival and beam balance test results.

15. Add scale bars to all microscopy images and indicate scale bar length in relevant figure legends.

Added. Thanks.

16. In general, figure legends have minimal information. They should be more descriptive.

As noted above, more information has been added. We did not include statistical information there in most cases because they were all the same and have been described in the Methods section.

Minor

1. Colour problems in bars of graphs are found in figures 3B, 3D, 5G, 5H, 6E, 6I, 6J.

The white or open bars for *Atg7* and *Palb2*;*Atg7* mice were purposely made white to distinguish them from the others, because most Purkinje cells had been lost at those time points and the counting was limited to the few remaining cells, which may also be dying. In the revised manuscript, we have removed these bars to avoid confusion. Thanks for your understanding.

2. In introduction, page 4, the phrase "In the current study, we aimed to co-delete Palb2 and Atg7 in the mammary gland using Cre recombinase driven by the promoter of whey acidic protein (Wap-Cre), to further study the role of autophagy in PALB2-associated breast cancer" could be misleading. Please rephrase, as breast cancer is not the main focus of the current study.

Thanks for a good suggestion. We have rephased the sentence as "In the current study, we originally aimed to…".

3. Missing in materials and methods: TH, p62, 4-HNE IHC staining information, Annexin V assay information. No γ-radiation methods are given. No cell doubling time measuring method for cell culture is provided.

Thanks for pointing these out. We have added these sections.

4. Regarding results of graphs in figures 7A and 7B, please explain them in more detail in the results text.

Please see new Figure 8 and text.

5. Indicate whether the stride result for WBKO animals presented in figure 1L represents fore or hind or both types of paws.

The result represents measurements of hind paws. This info has been added in the figure legends and methods.

6. Please reconsider the use of the word "condensed signal" to describe staining signal, in results page 10.

The word has been deleted.

7. The last sentence of paragraph "Requirement of PALB2 for mitochondrial homeostasis in the brain" describing MT-CO1 signal intensity for Palb2Δ/Δ mice should be moved right after the MT-CO1 data from the Atg7-CKO and Palb2;Atg7 double CKO mice in the same paragraph.

Moved as suggested.

8. In results, page 11, authors mention: "In keeping with our in vivo findings, MT-CO1, MT-CO2 and SOD2 were all increased in PALB2-KO, ATG7-KO and PALB2;ATG7-DKO cells, implying increased mitochondrial mass upon loss of either PALB2 or ATG7"

a. Please rephrase, as there are no data regarding MT-CO2 in the in vivo findings.

"In keeping with our in vivo findings" has been deleted. Thanks for pointing this out.

b. Please add reference correlating markers MT-CO1 and SOD2 with increased mitochondrial mass.

We do not have any such references. When we mentioned increased mitochondrial mass, our basis was a combination of increased MitoTracker Green signal and increased expression of MT-CO1, MT-CO2 and SOD2. In the revised manuscript, we have added RNA-seq data showing broad upregulation of mitochondrial genes upon PALB2 depletion (new Figure 4), providing further support for increased mitochondrial mass.

9. In results, page 15, change "rather than increased DNA damage, in the brain" to rather than increased DNA damage, in the cerebellum".

Changed. Thanks for the suggestion.

10. Add the full term of an abbreviation when used for the first time in the main text.

Added as suggested.

11. In introduction, page 3, and reference list, correct reference (Collaborators, 2019).

Corrected. Thanks.

12. Remove "n=4" from fig. 1E.

Removed. Thanks.

13. Correct I to L where appropriate in the legend of Figure 1.

Corrected. Thanks.

14. Correct “tyrosine hydrolase” to “tyrosine hydroxylase” in the relevant results section.

Corrected. Thanks.

15. Correct spelling and grammar mistakes such as “to expresses Cre” to “to express Cre”, “LC3-I o LC3-II” to “LC3-I to LC3-II”, “Supplementray” to “Supplementary”, “repiration” to “respiration” in results section, “neurodegenetation” to “neurodegeneration”, “acvtivation” to “activation” in the discussion section, e.t.c. Also “while” to “which” in discussion (page 16, the sentence regarding DCF-DA).

Spelling correction in MS Word didn’t work then. All corrected. Many thanks.

16. Change “While suppressing neurodegeneration…” to “Besides suppressing neurodegeneration…” in the introduction.

Changed. Thanks.
